# Resveratrol Suppresses Gut-Derived NLRP3 Inflammasome Partly through Stabilizing Mast Cells in a Rat Model

**DOI:** 10.1155/2018/6158671

**Published:** 2018-12-20

**Authors:** Weicheng Zhao, Xiaolei Huang, Xue Han, Dan Hu, Xiaohuai Hu, Yuantao Li, Pinjie Huang, Weifeng Yao

**Affiliations:** ^1^Department of Anesthesiology, The First People's Hospital of Foshan, 81 North of Rinlan Road, Foshan 528000, China; ^2^Department of Anesthesiology, Shenzhen Maternity and Child Healthcare Hospital, Southern Medical University, Shenzhen 518116, China; ^3^Department of Anesthesiology, Sun Yat-sen Memorial Hospital, Sun Yat-sen University, Guangzhou 510120, China; ^4^Department of Ophthalmology, The First People's Hospital of Foshan, 528000, China; ^5^Department of Medical Section, The First People's Hospital of Foshan, 528000, China; ^6^Department of Anesthesiology, The Third Affiliated Hospital of Sun Yat-sen University, Guangzhou 510630, China

## Abstract

**Background:**

Inflammatory responses induced by intestinal ischemia-reperfusion (IIR) lead to serious systemic organ dysfunction and pose a challenge for current treatment. This study aimed at investigating the effects of resveratrol on IIR-induced intestinal injury and its influence on mast cells (MCs) in rats.

**Methods:**

Rats subjected to intestinal ischemia for 60 min and 4 h of IIR were investigated. Animals were randomly divided into five groups (*n* = 8 per group): sham, IIR, resveratrol (RESV, 15 mg/kg/day for 5 days before operation) + IIR, cromolyn sodium (CS, MC membrane stabilizer) + IIR, and RESV + compound 48/80 (CP, MC agonist) + IIR.

**Results:**

Intestinal injury and increased proinflammatory cytokines including tumor necrosis factor-*α*, interleukin-1*β*, and interleukin-18 were observed in the IIR group. Intestinal MC-related tryptase and *β*-hexosaminidase levels were also increased after rats were subjected to IIR accompanied by activation of NLRP3 inflammasomes. Interestingly, pretreatment with resveratrol significantly suppressed the activities of proinflammatory cytokines and attenuated intestinal injury. Resveratrol also reduced MC and NLRP3 inflammasome activation, which was consistent with the effects of cromolyn sodium. However, the protective effects of resveratrol were reversed by the MC agonist compound 48/80.

**Conclusions:**

In summary, these findings reveal that resveratrol suppressed IIR injury by stabilizing MCs, preventing them from degranulation, accompanied with intestinal mucosa NLRP3 inflammasome inhibition and intestinal epithelial cell apoptosis reduction.

## 1. Background

As the intestine serves as a critical defense barrier in humans, attacks on intestinal barrier function may lead to the release of toxins from the enteric cavity [1]. Intestinal ischemia-reperfusion (IIR) is one of such life-threatening events that occurs in serious conditions such as acute mesenteric ischemia, shock, mesenteric thrombosis, sepsis, or bowel transplantation [2–5]. The natural barrier function of the intestine breaks down following IIR, which results in bacterial translocation, endotoxemia, and uncontrolled release of inflammatory mediators and cytokines. Next, a systemic inflammatory response and distant organ damage occurs [6, 7], followed by multiple organ dysfunction or multiple organ failure [8].

Recently, the inflammasome was identified as a critical checkpoint in inflammatory diseases [9]. To date, the NLR family pyrin domain-containing 3 (NLRP3) inflammasome is the most intensively studied inflammasome and has been recognized to play important roles in ischemia/reperfusion diseases [10]. Inflammasome activation causes maturation of cytokines such as interleukin (IL)-1*β* and IL-18 and induces a specific form of cell death known as pyroptosis [11]. Unlike apoptosis, pyroptosis requires caspase-1 activation and results in the pathogen-associated molecular pattern and proinflammatory cytokines release [11]. Pyroptosis has been suggested to be involved in various pathological and physiological conditions, particularly in the mucosal compartments including the gut [12]. However, the role of pyroptosis-related inflammasome activation in IIR injury remains unclear.

Resveratrol, a natural phytoalexin, [13], acts as a free radical scavenger and modulates several enzymes including cyclooxygenase, inducible nitric oxide synthase, lipoxygenase, and protein kinase C, which regulate cell life. [14]. Resveratrol is also recognized as a potent anti-inflammatory agent [15]. Previous studies showed that resveratrol prevented the lung from ischemia/reperfusion injury through mast cell- (MC-) dependent mechanisms in rats [16]; however, the downstream effects of MCs stabilized by resveratrol remain unclear.

We hypothesized that the downstream effects of MCs stabilized by resveratrol are mediated via NLRP3 inflammasome inhibition, which may effectively lead to intestinal barrier function restoration in the early stages. The present study aimed at investigating whether resveratrol pretreatment mitigates intestinal mucosa injury through suppressing NLRP3 inflammasome-related apoptosis by stabilizing MCs in a rat model of IIR injury.

## 2. Methods and Materials

### 2.1. Animals

Forty adult male Sprague-Dawley (SD) rats (220–250 g) were housed with three rats per cage in the experimental animal center of Sun Yat-sen University and were provided standard rat pellets as well as sterile water *ad libitum*. All procedures were approved by the authors and approved by the Institutional Animal Care and Use Committee at the Third Affiliated Hospital of Sun Yat-Sen University. This committee is guided by the Care and Use of Laboratory Animals (1996).

### 2.2. Induction of Intestinal Ischemic-Reperfusion Injury and Experimental Design

In the study, the 40 SD rats were assigned to five groups as follows:

1. Sham group: rats in the sham group underwent identical surgical interventions including laparotomy and vascular dissection without superior mesenteric artery clamping
2. IIR group: rats were anesthetized with 5% isoflurane in a sealed chamber (0.5 L/min O_2_). The abdomens were opened, and the superior mesenteric arteries were separated for clamping for 60 min with a small vessel clip, followed by 4 h of reperfusion
3. Resveratrol (RESV) + IIR group: resveratrol was administrated (15 mg/kg/day [17]) by intraperitoneal injection for 5 days prior to the IIR procedures
4. Cromolyn sodium (CS) + IIR group: cromolyn sodium was administrated via the tail vein at 25 mg/kg 15 min prior to the IIR procedures
5. RESV + compound 48/80 (CP) + IIR group: compound 48/80 (0.75 mg/kg) was injected into the tail vein 5 min before reperfusion

Continuous anesthesia was maintained on a 37°C heat mat. Animals were sacrificed by carbon dioxide asphyxiation (air in a suitable airtight container was displaced with carbon dioxide fed in from a compressed gas cylinder, and the animals were gently placed in the container for several minutes to ensure death). After 4 h reperfusion, blood samples were collected from the abdominal aorta and centrifuged at 3600 ×g for 15 min to isolate the serum. A segment of small intestine 10 cm away from the terminal ileum was removed and fixed in formalin. The remaining small intestine was collected immediately and stored at −80°C until analysis. The study has been performed more than once, and all the detection has been performed more than three biological replications.

### 2.3. Pathological Assessment

Paraffin-embedded intestinal sections (5 *μ*m) were used for hematoxylin and eosin staining (H&E) as previously described [18]. The degree of intestinal damage was scored using a classification previously described by Chiu et al. [19] on a scale from 0 to 5, which consists of five subdivisions based on changes to the villi and glands of the intestinal mucosa, as described in a previous study [20]. Grade 0 is the normal mucosa; grade 1 indicates the development of subepithelial Gruenhagen's space at the tip of the villus; grade 2 indicates the extension of the space with moderate epithelial lifting; grade 3 indicates massive epithelial lifting with a few denuded villi; grade 4 indicates denuded villi with exposed capillaries; grade 5 indicates disintegration of the lamina propria, ulceration, and hemorrhage.

### 2.4. Measurement of IL-1*β*, IL-18, TNF-*α*, and *β*-Hexosaminidase Levels

The methods used to measure cytokine levels were described in our previous study [21]. Rat serum was centrifuged and prepared to evaluate interleukin (IL)-1*β*, IL-18, and tumor necrosis factor (TNF)-*α* levels by enzyme-linked immunosorbent assay according to the manufacturer's instructions (KeyGEN Biotech Company, Nanjing, China). The optical absorbance was detected by using a microplate reader (Bio-Rad, Hercules, CA, USA). Intestinal *β*-hexosaminidase level was measured using a colorimetric method according to our previously described method [18].

### 2.5. Immunofluorescence Assay

Paraffin-embedded intestine blocks were sliced into 4 *μ*m sections. Tryptase (1 : 500, Santa Cruz Biotechnology, Dallas, TX, USA) staining was carried out to evaluate the expression of tryptase in MCs by using immunofluorescence methods. Sections were incubated with the tryptase primary antibody overnight at 4°C. A secondary antibody (1 : 200, Life Technologies, Carlsbad, CA, USA) was added for 1 hour at 37°C, and then the sections were rinsed with PBS three times. A microscope (DMLB2, Leica, Wetzlar, Germany) was used to observe the stained sections.

### 2.6. Terminal Deoxynucleotidyl Transferase-Mediated Nick End Labeling (TUNEL)

A TUNEL assay was performed to evaluate the cellular apoptosis of the injured intestine using the In Situ Cell Death Detection Kit (Roche, Basel, Switzerland) following the manufacturer's instructions.

### 2.7. Western Blot Assay

Intestinal tissue samples were homogenized and evaluated using the BCA method (Thermo Scientific, Waltham, MA, USA). Primary antibodies including anti-NLRP3 at a 1 : 100 dilution, anti-IL-1*β* p17 at a 1 : 1000 dilution, anti-caspase-1 p20 at a 1 : 1000 dilution, anti-IL-18 at a 1 : 1000 dilution, or anti-*β*-actin at 1 : 2000 dilution were used in western blot analysis, which was performed according to our previous studies [21].

### 2.8. Statistical Analysis

All data are shown as the mean values ± standard error of the mean. The software *SPSS 13.0* (SPSS, Inc., Chicago, IL, USA) was used for statistical analysis. Differences between multiple groups were compared by one-way analysis of variance followed by *Tukey's* post hoc test to compare the differences between individual groups. *P* < 0.05 was considered as statistically significant.

## 3. Results

### 3.1. Resveratrol Pretreatment Attenuated IIR Injury and Reduced Proinflammatory Cytokine Generation

**Figure 1:**
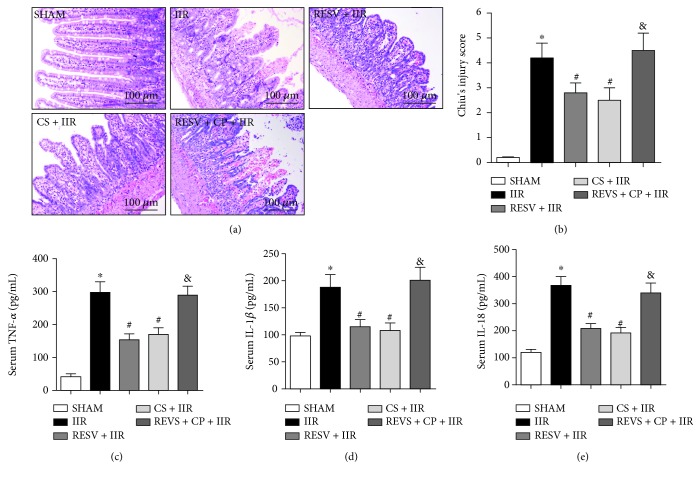
Resveratrol pretreatment attenuated IIR injury and reduced proinflammatory cytokine generation. (a) Representative histological illustration for each group, hematoxylin and eosin (H&E) staining, magnification: × 200. (b) *Chiu's* score. (c) Serum TNF-*α* activity. (d) Serum IL-1*β* activity. (e) Serum IL-18 activity. IIR: intestinal ischemia-reperfusion; RESV: resveratrol; CP: compound 48/80; CS: cromolyn sodium. Data are expressed as the means ± SEM, *n* = 8/group. ^∗^*P* < 0.05 vs. sham group, ^#^*P* < 0.05 vs. IIR group, ^&^*P* < 0.05 vs. RESV + IIR group.

As shown in Figure 1, no intestinal pathological changes were observed in the sham group. However, when tested at 4 h after reperfusion, IIR rats displayed severe intestinal injury (Figure 1(a)), evidenced by thinning of the intestinal mucosa and wall, loss and shedding of intestinal villi, and large numbers of inflammatory cells infiltrating the intestine, accompanied by an increase in proinflammatory cytokines, including TNF-*α*, IL-1*β*, and IL-18. Interestingly, in RESV + IIR or CS + IIR pretreatment groups, the villus arrangement was relatively regular, with no evidence of the loss of intestinal villi, and only small gaps formed at the top of the intestinal villi and few inflammatory cells were found in the intestine (Figure 1(a)). Additionally, both resveratrol and cromolyn sodium pretreatment significantly improved the Chiu's score and reduced the serum levels of the proinflammatory cytokines TNF-*α*, IL-1*β*, and IL-18 compared to the IIR group (*P* < 0.05) (Figures 1(b)–1(e)). However, compared to the RESV + IIR group, compound 48/80 treatment (RESV + CP + IIR) significantly increased *Chiu's* score and TNF-*α*, IL-1*β*, and IL-18 activities (*P* < 0.05), suggesting that the intestinal protective effects of resveratrol were reversed by the MC agonist compound 48/80.

### 3.2. Resveratrol Pretreatment Stabilized MC Activation

**Figure 2:**
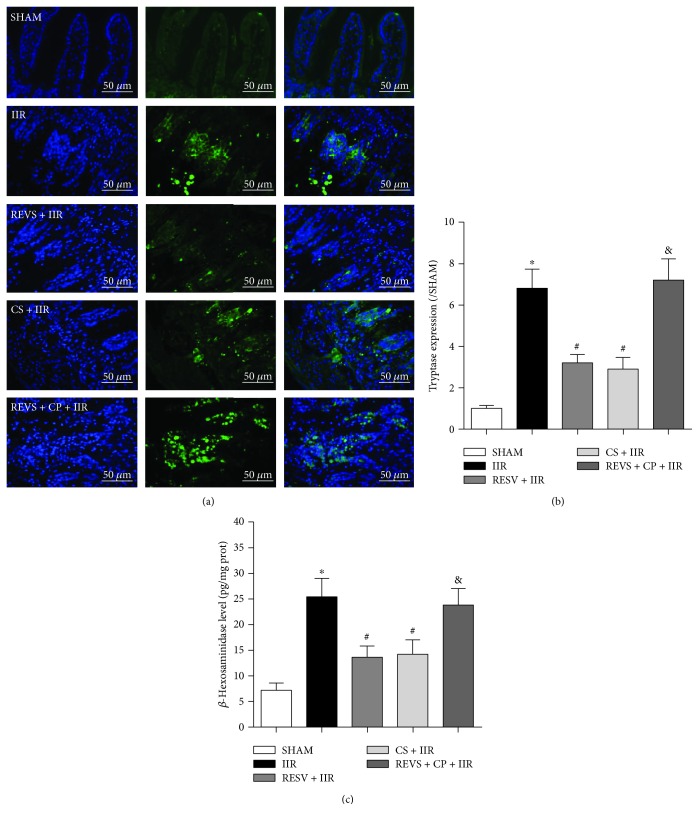
Resveratrol pretreatment stabilized mast cell activation. (a) Representative histological results for each group, tryptase immunofluorescence staining, magnification ×400. (b) Tryptase immunofluorescence staining gray value. (c) Intestinal *β*-hexosaminidase activity. IIR: intestinal ischemia-reperfusion; RESV: resveratrol; CP: compound 48/80; CS: cromolyn sodium. Data are expressed as the means ± SEM, *n* = 8/group. ^∗^*P* < 0.05 vs. sham group, ^#^*P* < 0.05 vs. IIR group, ^&^*P* < 0.05 vs. RESV + IIR group.

To further investigate whether intestinal protective effects were associated with MC activation, the MC membrane stabilizer cromolyn sodium and MC agonist compound 48/80 were used in *in vivo* experiments, and MC markers were detected. As shown in Figures 2(a)–2(c), level of the MC markers tryptase and *β*-hexosaminidase was increased in the intestine after rats were subjected to IIR. Resveratrol pretreatment significantly reduced tryptase expression and *β*-hexosaminidase activities in the intestine (*P* < 0.05, vs. IIR group). These results were similar to those observed in the CS + IIR group, suggesting that the intestinal protective effects of resveratrol are related to MC stabilization. Furthermore, in the REVS + CP + IIR group, compound 48/80 treatment reversed the MC stabilizing effects evidenced by decreases in tryptase expression and *β*-hexosaminidase activities. These results indicate that the anti-inflammatory effects of resveratrol on IIR injury were closely correlated with MC stabilization.

### 3.3. Resveratrol Pretreatment Inhibited NLRP3 Inflammasome Activation by Stabilizing MCs

**Figure 3:**
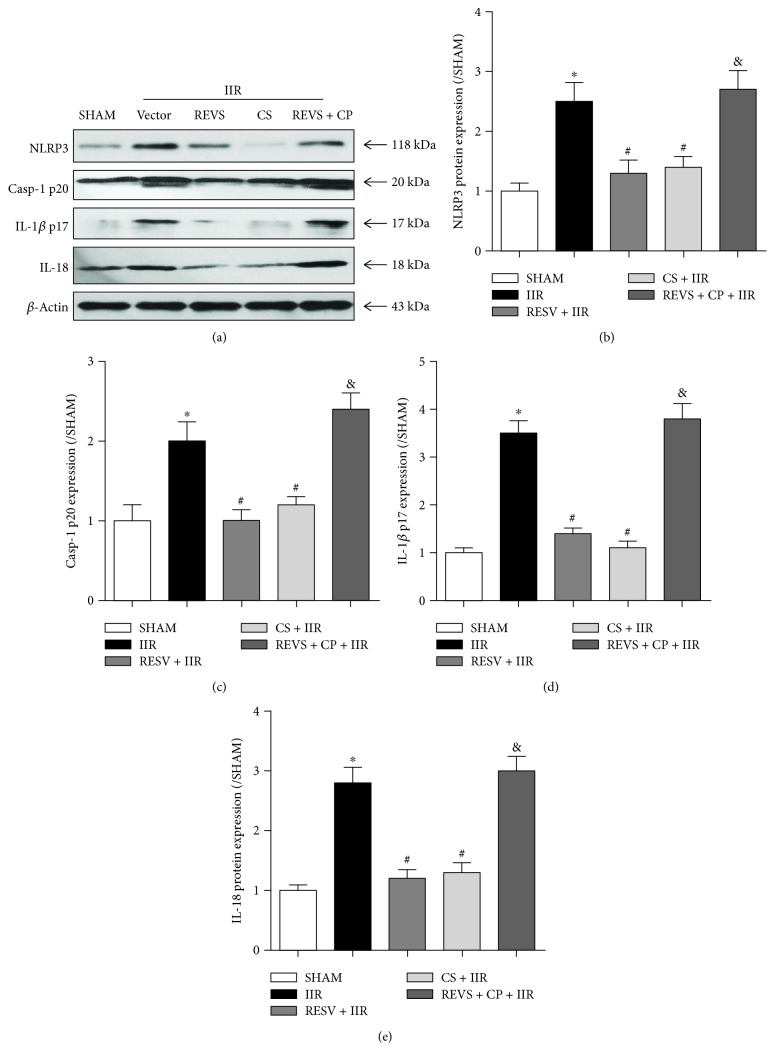
Resveratrol pretreatment inhibited NLRP3 inflammasome activation by stabilizing mast cells. (a) Representative western blotting bands of NLRP3, caspase-1 p20, IL-1*β* p17, and IL-18 for each group. (b) NLRP3 protein expression level. (c) Caspase-1 p20 protein expression level. (d) IL-1*β* p17 protein expression level. (e) IL-18 protein expression level. IIR: intestinal ischemia-reperfusion; RESV: resveratrol; CP: compound 48/80; CS: cromolyn sodium. Data are expressed as the means ± SEM, *n* = 8/group. ^∗^*P* < 0.05 vs. sham group, ^#^*P* < 0.05 vs. IIR group, ^&^*P* < 0.05 vs. RESV + IIR group.

Because IL-1*β* and IL-18 levels were reported to be increased following NLRP3 inflammasome activation [22], we further evaluated intestinal mucosal protein expression of NLRP3, caspase-1 p20, IL-1*β* p17, and IL-18. We found an increased mucosal protein expression of these proteins in rats subjected to IIR (*P* < 0.05, vs. sham group) (Figures 3(a)–3(e)). Resveratrol or cromolyn sodium pretreatment significantly decreased mucosal protein expression of the same proteins (*P* < 0.05, vs. IIR group) (Figures 3(a)–3(e)). However, the inhibitory effects of resveratrol on NLRP3 inflammasome levels were reversed by the MC agonist compound 48/80, as evidenced by significant increases in mucosal protein expression of NLRP3, caspase-1 p20, IL-1*β* p17, and IL-18 in the REVS + CP + IIR group (*P* < 0.05, vs. RESV + IIR group) (Figures 3(a)–3(e)).

### 3.4. Resveratrol Pretreatment Reduced Intestinal Cellular Apoptosis

**Figure 4:**
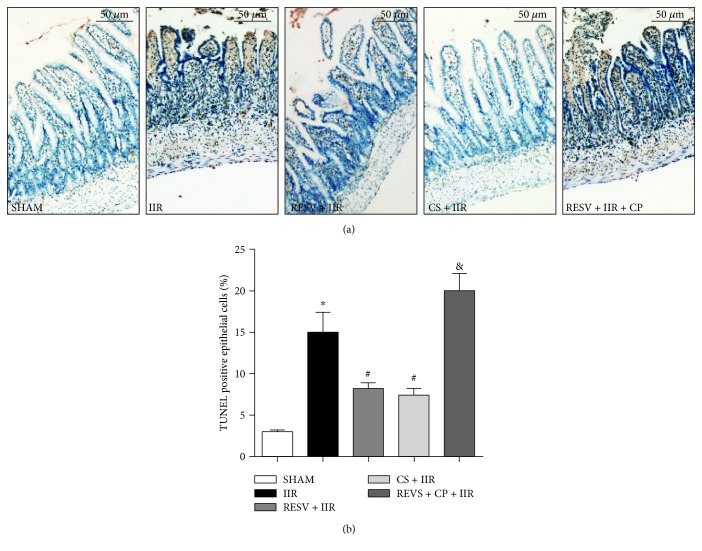
Resveratrol pretreatment reduced intestine cellular apoptosis. (a) Representative histological illustration for each group, TUNEL staining, magnification: ×400. (b) TUNEL-positive cell ratio. IIR: intestinal ischemia-reperfusion; RESV: resveratrol; CP: compound 48/80; CS: cromolyn sodium. Data are expressed as the means ± SEM, *n* = 8/group. ^∗^*P* < 0.05 vs. sham group, ^#^*P* < 0.05 vs. IIR group, ^&^*P* < 0.05 vs. RESV + IIR group.

As NLRP3 inflammasome activation induces cellular apoptosis [23], we next investigated intestinal apoptosis to explore the effects of resveratrol on IIR injury and of its effects on reducing intestinal cellular apoptosis. As shown in Figure 4, we found that IIR evoked intestinal cellular apoptosis detected as increased levels of TUNEL-positive cells (Figures 4(a) and 4(b)). Resveratrol (RESV + IIR) or cromolyn sodium (CS + IIR) pretreatment significantly reduced the number of TUNEL-positive cell numbers compared in the IIR group (*P* < 0.05). However, in the REVS + CP + IIR group, compound 48/80 treatment reversed the antiapoptosis effects conferred by resveratrol evidenced as decreased numbers of TUNEL-positive cells.

## 4. Discussion

IIR, with high clinical morbidity and mortality, has been identified as a common and devastating condition [7] that aggressively damages the mucosal barrier function [6, 24] and for which preventive interventions are still lacking. Our findings indicate that MC activation plays a critical role in IIR injury by breaking down intestinal barrier integrity with subsequent triggering of proinflammatory cytokine release and cellular apoptosis. However, resveratrol pretreatment suppressed intestine-derived NLRP3 inflammasome-related pyroptosis by stabilizing and preventing MCs from degranulation.

Restoration of blood flow following a period of intestinal ischemia is an inevitable process [25]; however, reintroduction of oxygen initiates a cascade of harmful events that exacerbate intestinal injury [26, 27]. IIR injury is manifested as increased microvascular and mucosal permeability and mucosal cellular apoptosis [28]. Recently, we found that gut-derived MC activation occurred during the reperfusion stage and that stabilizing MCs effectively attenuated IIR injury [29]. Interestingly, in the present study, we found that resveratrol pretreatment reduced gut-derived MCs and stabilized and prevented MCs from degranulation, resulting in the restoration of damaged intestine barrier function. Our results agree with those of Shirley et al. [30] who showed that resveratrol exerts anti-inflammatory properties at low concentrations by targeting the arachidonic acid (AA) pathway and elevates TNF-*α* generation from human mature MCs in an IgE-dependent manner. This indicates that the intestinal protective effects of resveratrol occur through interactions with MCs. However, except for inhibition of TNF-*α* release, we also found that resveratrol reduced the production of tryptase which was dramatically increased in the intestinal mucosa after MC activation, indicating that resveratrol functions as a MC stabilizer during injury.

Previous studies suggested that resveratrol plays therapeutic roles in ischemia/reperfusion-related diseases [31–34]. Resveratrol shows a broad spectrum of protective effects by increasing the antioxidative capacity and reducing the oxidative status of target organs during IIR injury [35–39]. In the present study, we found that resveratrol suppressed the activities of the proinflammatory cytokines IL-18, IL-1*β*, and TNF-*α*, indicating that it has potent anti-inflammatory properties. Similarly, a previous study revealed that in the blood of melanoma cell-infiltrated livers, TNF-*α* and IL-1*β* were inhibited, and IL-18 augmentation was prevented by treatment of resveratrol. The cytokines IL-18 and IL-1*β* are regulated by inflammasomes, which are composed of cytosolic protein complexes [40]. Intracellular caspase-1 is activated by inflammasomes, resulting in cleavage of the inactive precursors IL-1*β* and IL-18 to bioactive cytokines [41]. To date, three inflammasome form NLRP1, NLRP3, and NLRC4 have been found to contain a member of the intracellular Nod-like receptor (NLR) family [42]. The NLRP3 inflammasome is activated by multiple stimuli including adenosine triphosphate, particulate matter, and microbial RNA [10]. In our study, we found that MC activation was associated with NLRP3 inflammasome activation and that resveratrol-inhibited NLRP3 inflammasome activation by stabilizing MCs from degranulation. MC degranulation was reported to be closely related to antigen-crosslinking of IgE bound to the receptor Fc*ε*RI on MCs [43]. Resveratrol reduced allergen-specific IgE levels and prevented MCs from responding to IgE [44]. However, in the current study, we found that the effects of resveratrol on MCs were more similar to those of cromolyn sodium which stabilizes MCs by regulating calcium flux [45].

Notably, although NLRP3 inflammasomes were found inhibited via MC inhibition in the current study, the underlying mechanisms remain unknown. Whether other cell types also affect NLRP3 inflammasome activation during IIR requires further investigation. Moreover, in the current study, resveratrol was administered before rats were subjected to IIR injury, whereas in IIR-related clinical settings, it is not possible to predict the onset of ischemia. Whether resveratrol is administered during or after IIR requires further investigation.

## 5. Conclusions

**Figure 5:**
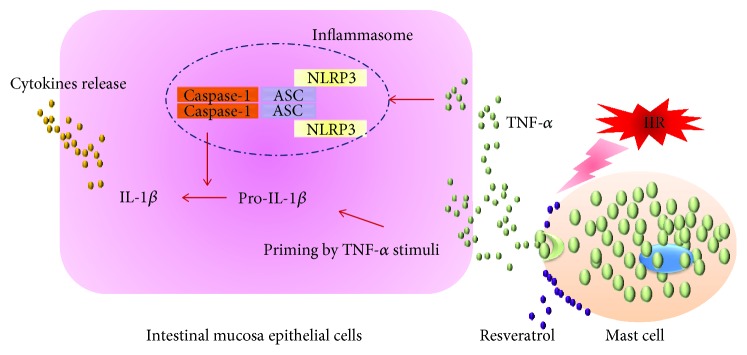
Resveratrol reduces intestinal ischemia-reperfusion injury by inhibiting mast cell-related NLRP3 inflammasome activation. Under intestinal ischemia-reperfusion conditions, mast cells are activated and degranulated. Cytokine TNF-*α* was released following mast cells activation, resulting in NLRP3-related inflammasome activation and subsequent IL-1*β* generation. Amount of proinflammatory cytokines produced and released from intestinal mucosa epithelial cells. Resveratrol pretreatment prevented the mast cells from degranulation via stabilizing mast cell membrane, which inhibited intestinal mucosa epithelial cell NLRP3-related inflammasome activation.

In summary, our findings reveal an intestinal protective role for resveratrol in IIR injury. Pretreatment with resveratrol restores the intestinal mucosal barrier, inhibits MC and NLRP3-inflammasome activation, and limits proinflammatory cytokine release. These results strongly suggest that inhibition of MC-related inflammasome activation is a promising strategy for preventing the deleterious effects of various conditions associated with intestinal ischemia and reperfusion (Figure 5).

## Data Availability

The data used to support the findings of this study are available from the corresponding author upon request.
